# Intraneural Ganglion Cyst of the Tibial Nerve

**DOI:** 10.5334/jbr-btr.860

**Published:** 2015-09-15

**Authors:** R. Vandenbulcke, J. Marrannes, B. Vandenbulcke, M. Herman, E. Laridon, B. Van Holsbeeck

**Affiliations:** 1 Department of Radiology, AZ Delta Campus Brugsesteenweg, Roeselare, Belgium; 2 General practitioner, Ingelmunster, Belgium

A 30-year-old marathon runner presented with intermittent posterior right knee pain. Symptoms had initiated after a long training session two months previously. On physical examination the popliteal fossa was sensitive to direct compression but no muscular weakness or neurological deficits were noted. His medical history was unremarkable. Radiographs of the right knee revealed no significant abnormalities and treatment with non-steroidal anti-inflammatory drugs showed no noteworthy improvement. MRI examination demonstrated a multilocular hyperintense mass on T2 weighted imaging (WI) along the course of the tibial nerve in the popliteal fossa (Fig. A1). 3D Reconstruction showed extension of this lesion anteriorly into the muscular branches of the popliteus muscle and into an articular branch to the superior tibiofibular joint (Fig. A2). This cystic lesion caused an eccentric displacement of the nerve fascicles (signet ring sign) (Fig. B1). Appearing hypointense on T1 WI, there was no enhancement of the mass after contrast study (Fig. B2). Slightly increased T2 signal was noted in the popliteus muscle belly indicating denervation edema (Fig. C). Based on these MRI-findings, diagnosis of a tibial intraneural ganglion cyst was made.

**Figures A–C:**
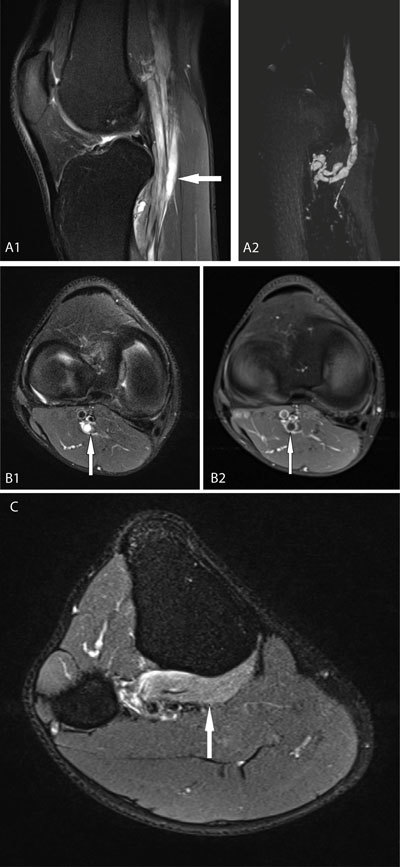


## Comment

Intraneural ganglion cysts of the tibial nerve are extremely rare with only few cases reported in literature. The common peroneal nerve is most frequently involved and other affected nerves are often located near articular spaces. These cystic lesions, filled with mucinous fluid and encased with a fibrous wall, are located within the epineurium causing an eccentric displacement of the nerve fascicles. Main clinical symptoms include posterior knee pain, motor weakness and paresthesia. Various explanations have been postulated about the pathogenesis of these ganglia, but based on recent clinic-anatomical findings and MR imaging, the ‘unified articular theory’ is the most supported. Hereby several authors are suggesting a dissection of synovial joint fluid through capsular defects via small articular branches and migrating up along a path of least resistance in the epineurium of a major nerve. Trauma is considered as a contributing factor rather than a principal causative factor of this entity. Although these lesions could be recognized on ultrasonography, MRI appears to be the modality of choice to evaluate the anatomical relation of the cyst to the joint and the surrounding structures. MRI helps differentiating intraneural ganglion cysts from other similar lesions such as cystic schwannoma (solid internal tumor enhancing components), myxoma or synovial sarcoma. Baker cysts have similar signal characteristics but are located between the medial head of the gastrocnemius and the semimembranosus tendon. Minimally invasive ultrasound-guided aspiration techniques are described but potential shortcomings include cyst recurrence, infection and fascicular or vessel injury. Surgical treatment is preferential for symptomatically patients refractory to minimally invasive percutaneous treatment. Decompression of the intraneural ganglion cyst, synovial resection of the approximate joint and especially eliminating the causative articular branch during an open microsurgical approach is providing satisfactory long term results and low recurrence rates.

## Competing Interests

The authors declare that they have no competing interests.
